# Comparative effectiveness and safety of Huangdi internal needling versus ibuprofen for primary dysmenorrhea

**DOI:** 10.1097/MD.0000000000049732

**Published:** 2026-08-21

**Authors:** Jing Liu, Wei Wang, Zun Liu

**Affiliations:** a Health and Wellness Industry-Education Integration Training Center, Cangzhou Medical College, Cangzhou City, Hebei Province, China.

**Keywords:** acupuncture, adverse events, Cox Menstrual Symptom Scale, Huangdi internal needling, ibuprofen, primary dysmenorrhea, retrospective cohort study, Visual Analog Scale

## Abstract

Primary dysmenorrhea (PDM) is highly prevalent among reproductive-aged women and is commonly managed with nonsteroidal anti-inflammatory drugs (NSAIDs), although medication-related adverse events and incomplete symptom control remain clinically relevant. Huangdi internal needling (HIN) is a traditional acupuncture-based technique that has been applied in pain conditions, but real-world comparative data in PDM are limited. This retrospective cohort study reviewed consecutive PDM patients treated at a single center between July 2021 and September 2023. Patients receiving HIN were compared with those managed using ibuprofen sustained-release capsules. Pain intensity and menstrual symptom burden were assessed using the Visual Analog Scale (VAS) and the Cox Menstrual Symptom Scale (CMSS) at baseline, after each of 3 menstrual cycles, and at a 6-month follow-up. Safety was evaluated by documented adverse events graded using CTCAE criteria. A total of 132 patients were included (66 per group). Baseline characteristics were comparable. Across treatment cycles and at 6 months, the HIN group demonstrated lower mean VAS scores than the ibuprofen group (baseline: 7.63 vs 7.78; cycle 3: 1.58 vs 3.24; 6 months: 2.36 vs 4.22; all between-group *P* < .05). CMSS scores also favored HIN at each posttreatment assessment. No adverse events were recorded in the HIN group, whereas 9 patients in the ibuprofen group reported grade 1 gastrointestinal symptoms that resolved after drug discontinuation. In routine clinical practice, HIN was associated with greater symptom improvement and fewer adverse events than ibuprofen for PDM, supporting further validation in multicenter studies.

## 1. Introduction

Primary dysmenorrhea (PDM) is mainly seen in adolescent or unmarried women, characterized by periodic lower abdominal pain before and after menstruation or the menstrual period, accompanied by lumbosacral pain, but without organic lesions of the reproductive system. PDM mostly occurs in adolescent girls or childless young women within 2 to 3 years after menarche.^[1,2]^ The prevalence of PDM in menstruating women is 45% to 72%.^[3]^ Western medicine primarily relies on non-narcotic analgesics and nonsteroidal anti-inflammatory drugs (NSAIDs) to manage PDM, which demonstrate some efficacy but are associated with significant limitations.^[4,5]^ Reports indicate that NSAIDs may cause adverse effects such as digestive tract symptoms, headaches, and drowsiness. In contrast, Chinese medicine offers substantial advantages in the treatment of PDM.^[6]^ As an alternative therapy, acupuncture has become an important method to treat pain. Clinical studies have demonstrated that acupuncture therapy not only alleviates pain in patients but also mitigates associated symptoms of menstrual discomfort, including emotional depression.^[7,8]^ However, there are many ways to use acupuncture in clinical practice; for example, the selected acupoints are different, so this treatment method still lacks standardization.^[9,10]^ Therefore, the selection of standardized and effective acupoints is very important for the analgesic effect of acupuncture on PDM. Huangdi internal needle, an acupuncture therapy originating from Huangdi internal needle, has a long history and wide recognition.^[11]^ In this study, Huangdi internal acupuncture therapy was employed to treat PDM patients over 3 treatment cycles.

## 2. Materials and methods

### 2.1. Research design

This study was approved by the Ethics Committee of Cangzhou Medical College. This single-center, retrospective cohort study included consecutive patients with primary dysmenorrhea (PDM) who were treated at our hospital between July 2021 and September 2023. Eligible cases were identified through a systematic review of electronic medical records, including outpatient/inpatient clinical notes, treatment orders, and follow-up documentation. Patients were classified into the Huangdi Internal Needling group or the ibuprofen group according to the actual intervention received in routine clinical practice. Because this was a retrospective observational study, treatment allocation was not randomized. Therefore, potential selection bias and residual confounding could not be completely excluded. Pain intensity and menstrual symptom burden were evaluated using the Visual Analog Scale (VAS) and the Cox Menstrual Symptom Scale (CMSS) at baseline and at prespecified follow-up time points documented in the records (across 3 menstrual cycles and at 6 months). Safety was assessed by extracting adverse event information from clinical documentation and grading severity using the Common Terminology Criteria for Adverse Events (CTCAE). The study was conducted in accordance with the Declaration of Helsinki and was approved by the institutional ethics committee. Written informed consent was obtained from all participants prior to treatment and follow-up evaluations as part of routine clinical care, and consent records were retained in the medical charts; participants were informed of the treatment procedures, potential risks, and their right to withdraw at any time.

### 2.2. Inclusion and exclusion criteria

#### 2.2.1. Inclusion criteria

Patients fulfill the following criteria: women aged 18 to 30 with no history of childbirth; B-ultrasound examination did not reveal significant organ lesions; diagnosis of PDM according to the Consensus Guidelines for Primary Dysmenorrhea^[12,13]^; regular menstrual cycles (28 ± 7 days) during the first 3 cycles; a Visual Analog Scale (VAS) average score >4 cm (from 0 to 10 cm); voluntary signing of informed consent for participation and agreement to undergo every research technique.

#### 2.2.2. Exclusion criteria

Among the exclusion criteria were: Diagnosis of secondary dysmenorrhea due to polycystic ovary syndrome or endometriosis confirmed by B-ultrasound and gynecological examination^[14]^; patients with serious conditions such as central nervous system disorders, mental illness, cardiovascular and cerebrovascular diseases, or impaired liver and kidney function; patients with skin infections or spontaneous bleeding disorders; women who were pregnant, lactating, or preparing for pregnancy; participants currently involved in other clinical trials.

### 2.3. Treatment measures

#### 2.3.1. The observation group (HIN group)

The observation group (HIN Group) received Huangdi internal acupuncture therapy. The acupuncture protocol is as follows:

1. Basis for acupoint selection: The 7 meridians traversing the human abdomen are as follows: the Conception Vessel (Ren Mai) runs along the midline. The Kidney Meridian of Foot-Shaoyin (Zu Shao Yin Shen Jing) runs bilaterally, 0.5 cun lateral to the midline. The Stomach Meridian of Foot-Yangming (Zu Yang Ming Wei Jing) runs bilaterally, 2 cun lateral to the midline. The Spleen Meridian of Foot-Taiyin (Zu Tai Yin Pi Jing) runs bilaterally, 4 cun lateral to the midline.
2. Acupoint selection for acupuncture: Based on meridian and acupoint theory, Lieque (LU7) is an acupoint of the Lung Meridian of Hand-Taiyin (Shou Tai Yin Fei Jing). The Lung Meridian of Hand-Taiyin and the Spleen Meridian of Foot-Taiyin are corresponding meridians sharing the same name (Taiyin) and thus have an affinity. Furthermore, Lieque (LU7) connects with the Conception Vessel. Shenmen (HT7) is an acupoint of the Heart Meridian of Hand-Shaoyin (Shou Shao Yin Xin Jing). The Heart Meridian of Hand-Shaoyin and the Kidney Meridian of Foot-Shaoyin are corresponding meridians sharing the same name (Shaoyin) and thus have an affinity. Yangxi (LI5) is an acupoint of the Large Intestine Meridian of Hand-Yangming (Shou Yang Ming Da Chang Jing). The Large Intestine Meridian of Hand-Yangming and the Stomach Meridian of Foot-Yangming are corresponding meridians sharing the same name (Yangming) and thus have an affinity. Guided by the principle of “treating the right side for left-sided disorders and vice versa” from the Huangdi Neizhen (Yellow Emperor’s Internal Needling), with the specific rule of selecting the right side for midline conditions, acupoints were selected according to the patient’s pain location. For severe pain at the midline of the lower abdomen, the right Lieque (LU7) was selected. For pain 0.5 cun lateral to the midline of the lower abdomen, the contralateral Shenmen (HT7) was selected. For severe pain 2 cun lateral to the midline of the lower abdomen, the contralateral Yangxi (LI5) was selected. For severe pain 4 cun lateral to the midline of the lower abdomen, the contralateral Lieque (LU7) was selected.
3. Acupuncture procedure: Following routine skin disinfection, stainless steel filiform needles (Hua Tuo brand, 0.25 mm × 25 mm, Suzhou Medical Supplies Co., Ltd.) were used. At Lieque (LU7), the needle was inserted obliquely 0.5 cun towards the elbow. At Shenmen (HT7), the needle was inserted perpendicularly 0.3 cun. At Yangxi (LI5), the needle was inserted perpendicularly 0.5 cun. No reinforcing or reducing techniques, such as lifting, thrusting, or rotating, were applied. The needles were retained for 30 minutes. During needle retention, patients were guided to focus their attention on the affected area and perceive the needling sensation (e.g., soreness, numbness, distension, heaviness). Acupuncture treatment was administered at the onset of dysmenorrhea and concluded upon pain relief. Therapeutic efficacy was evaluated after 3 menstrual cycles. The acupuncture procedures are overseen by a senior acupuncturist with more than 5 years of experience, ensuring treatment standardization and consistency within our hospital’s acupuncture department.

#### 2.3.2. The ibuprofen group was treated with ibuprofen

Patients in the Ibuprofen group received treatment with ibuprofen sustained-release capsules (300 mg/capsule, produced by Sino-American Tianjin Shike Pharmaceutical Co., Ltd., approval number: H10900089). The capsules were taken every 12 hours starting 5 days before the anticipated menstrual period until the cessation of menstruation on the first day. This treatment regimen was repeated over 3 consecutive menstrual cycles.

### 2.4. Observation index

#### 2.4.1. Visual Analogue Scale of pain score (VAS)

The VAS is a 10 cm sliding scale where “0” represents no pain at the leftmost end, and “10” signifies the most severe and unbearable pain at the rightmost end. During assessment, patients indicate their perceived pain intensity by marking a point along the unnumbered side of the scale, which is then read against the numbered side for scoring. Higher scores indicate more intense pain. Throughout the treatment, patients received acupuncture at the onset of pain during each menstrual cycle, with the entire treatment spanning 3 menstrual cycles. VAS scores represented the maximum pain intensity experienced during the first 24 hours of menstruation and were recorded at baseline, after each of the first 3 treatment cycles, and at the 6-month follow-up visit.^[15]^

#### 2.4.2. Cox Menstrual Symptom Scale (CMSS)

The scale encompasses 18 items assessing symptoms such as abdominal pain, headache, low back pain, stomachache, leg pain, nausea, vomiting, loss of appetite, fatigue, dizziness, diarrhea, complexion change, facial flushing, insomnia, general pain, depression, irritability, and nervousness. Each symptom’s total duration and average severity were evaluated, categorized into 5 grades: 0 (no pain), 1 (mild symptoms, noticeable but not hindering daily activities), 2 (moderate symptoms, not significantly affecting daily activities), 3 (pronounced symptoms, impacting daily activities), and 4 (severe symptoms, severely affecting daily activities). Pain duration was scored as follows: 0 (no pain), 1 (<3 hours), 2 (3–7 hours), and 3 (>7–24 hours). Before treatment, patients reviewed the average severity and duration of pain over the previous 3 menstrual cycles for evaluation. Short-term and long-term efficacy were assessed using CMSS scores at the end of each treatment cycle and at a 6-month follow-up.^[16]^

#### 2.4.3. Evaluation of treatment safety

Various adverse reactions occurring in patients from both groups during treatment were analyzed, and the Common Terminology Criteria for Adverse Events (CTCAE) were used to rate their severity,^[17]^ which includes 6 grades. Grade 1 indicates mild adverse reactions, characterized by mild symptoms that resolve after discontinuation of treatment. Grade 2 signifies moderate adverse reactions, resulting in temporary patient discomfort requiring treatment or intervention, but with a high likelihood of recovery.

Grades 3 to 6 denote severe adverse reactions:

1. Grade 3 indicates significant but temporary harm to the patient, requiring hospitalization or a prolonged hospital stay for outpatient management.
2. Grade 4 denotes permanent damage, such as disabling or irreversible organ damage.
3. Grade 5 indicates life-threatening reactions, such as shock or respiratory failure.
4. Grade 6 indicates death.

These grades provide a standardized framework for assessing and categorizing the severity of adverse events encountered during the study.

### 2.5. Statistical analysis

With IBM’s SPSS (Statistical Package for the Social Sciences) 23.0 program, all experimental data were statistically analyzed. Measurement results with homogenous variance and a normal distribution were reported as ($\bar{x}\pm s$). To compare the 2 groups, an independent sample *t* test was used. The statistical data were represented using the intragroup paired *t* test with n (%) and the χ^2^ test. A statistically significant difference was shown by a *P*-value of <.05.

## 3. Results

### 3.1. Comparison of general data between 2 groups of patients

Initially, 136 patients in all were included in this study. In the HIN group, 1 patient withdrew due to personal reasons, and another patient was excluded due to receiving other treatments. In the ibuprofen group, 2 patients withdrew for personal reasons. As a result, there were 66 patients remaining in both the HIN group and the ibuprofen group for analysis. There were no differences involving the 2 groupings that were statistically significant in baseline characteristics such as age, age at menarche, menstrual cycle length, duration of dysmenorrhea, body mass index (BMI), physical fitness group (ECOG), normal coagulation function, and absence of infection (*P* > .05). This suggests that the baseline characteristics of the 2 groups were similar (Table 1).

**Table 1:** Baseline characteristics of 2 groups of patients collected on the first day of the intervention cycle.

Groups	Observation group (N = 66)	Control group (N = 66)	*t*	*P* value
Age (yr)	22.63 ± 4.73	22.89 ± 4.65	0.318	.751
Age of menarche (yr)	13.96 ± 1.26	14.01 ± 1.45	0.211	.833
Menstrual cycle (d)	29.02 ± 3.65	28.87 ± 3.45	0.243	.809
Menstruation (d)	6.56 ± 1.01	6.77 ± 0.99	1.206	.230
Duration of dysmenorrhea (yr)	4.35 ± 0.65	4.52 ± 0.71	1.435	.154
BMI (kg/m^2^)	23.48 ± 2.13	23.40 ± 2.19	0.213	.832
ECOG grade (points)	2.13 ± 0.55	2.17 ± 0.57	0.410	.682
Is the coagulation function normal?	All normal	All normal		
Is there an infection?	No infection	No infection		

ECOG = Eastern Cooperative Oncology Group.

### 3.2. Comparison of VAS scores of each node involving the 2 groupings before and after treatment

In this study, VAS scores were used to evaluate dysmenorrhea treatment efficacy in 132 patients (66 in the HIN group and 66 in the ibuprofen group). Before treatment, the VAS scores were (7.63 ± 2.35) points for the HIN group and (7.78 ± 2.18) points for the Ibuprofen group, with no significant difference (*P* > .05). At the end of the first cycle, the VAS score decreased to (4.75 ± 1.34) points in the HIN group and (5.34 ± 1.57) points in the ibuprofen group, with a statistically significant difference involving the groupings (*P* < .05). After the second cycle, the HIN group’s score further decreased to (3.66 ± 0.87) points, while the ibuprofen group’s score was (4.59 ± 0.91) points, also demonstrating a notable change (*P* < .001). After the third treatment cycle, the HIN group had a mean VAS score of (1.58 ± 1.33) points compared to (3.24 ± 1.21) points in the ibuprofen group, with a notable variation involving the groupings (*P* < .001).

Six months after treatment completion, the HIN group had a VAS score of (2.36 ± 1.63), whereas the ibuprofen group had a score of (4.22 ± 1.73). There was a statistically significant difference (*P* < .001). These findings suggest that Huangdi internal acupuncture therapy significantly reduced pain intensity compared to ibuprofen treatment, both during the treatment period and in the long-term follow-up assessment (Table 2).

**Table 2:** Comparison of VAS scores of each node involving the 2 groupings before and after treatment ($\bar{x}\pm \;s$, points).

Groups	Observation group (N = 66)	Control group (N = 66)	*t*	*P* value
Before treatment	7.63 ± 2.35	7.78 ± 2.18	0.380	.704
First cycle treatment	4.75 ± 1.34	5.34 ± 1.57	2.322	.022
Second cycle treatment	3.66 ± 0.87	4.59 ± 0.91	6.001	<.001
Third cycle treatment	1.58 ± 1.33	3.24 ± 1.21	7.500	<.001
Six months after the end of treatment	2.36 ± 1.63	4.22 ± 1.73	6.357	<.001

VAS = Visual Analog Scale.

### 3.3. Comparison of CMSS score of COX menstrual symptom scale between 2 groups

**Figure 1.**
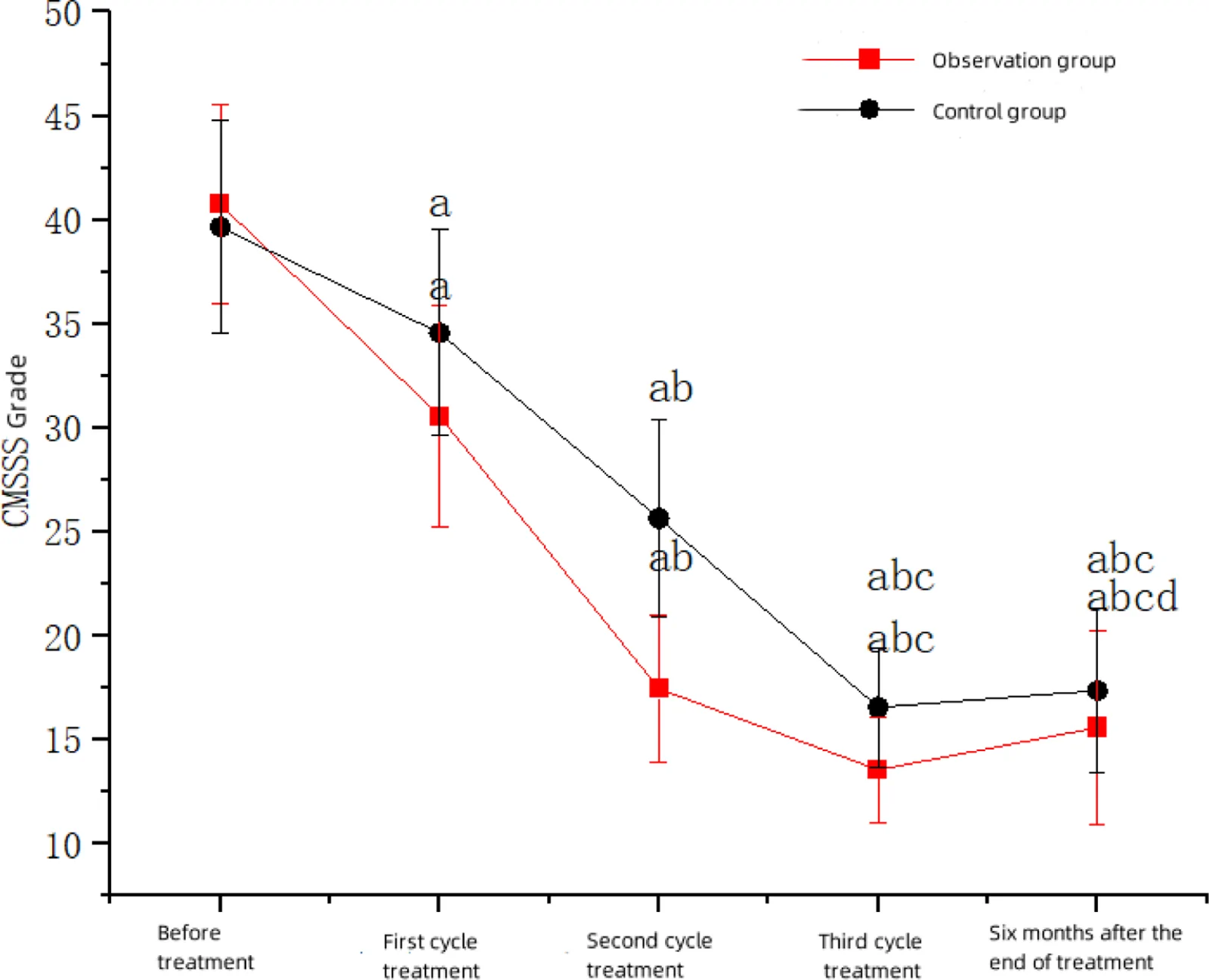
CMSS scores of each node in 2 groups before and after treatment. Note: a is *P* < .05 compared to the same group’s pretreatment; b is *P* < .05 compared to the group’s first cycle of treatment; c is *P* < .05 compared to the group’s second cycle of medication; and d is *P* < .05 compared to the group’s third cycle of treatment. CMSS = Cox Menstrual Symptom Scale.

In this study, CMSS scores were used to assess changes in menstrual symptoms in 132 patients (66 in the HIN group and 66 in the ibuprofen group) before and after treatment. Before treatment, the average CMSS score was (40.75 ± 4.79) points for the HIN group and (39.65 ± 5.13) points for the ibuprofen group, without a discernible change involving the 2 groupings (*P* > .05). After the first cycle, the CMSS score decreased to (30.54 ± 5.35) points in the HIN group and (34.56 ± 4.95) points in the Ibuprofen group, demonstrating a considerable change (*P* <.001). In the second cycle, the HIN group’s CMSS score was (17.43 ± 3.56) points compared to (25.64 ± 4.78) points in the Ibuprofen group, with a notable distinction (*P* < .001). By the end of the third treatment cycle, the HIN group had a CMSS score of (13.54 ± 2.56) points, whereas the ibuprofen group’s score was (16.54 ± 2.89) points, demonstrating a notable change (*P* < .001). Six months after treatment completion, the HIN group had a CMSS score of (15.57 ± 4.68) points, while the Ibuprofen group’s score was (17.35 ± 3.93) points. This difference remained statistically significant (*P* < .05, Fig. 1).

During the entire trial period, no adverse events were reported among patients in the HIN group receiving Huangdi internal acupuncture therapy. In contrast, in the Ibuprofen group receiving ibuprofen treatment, 9 patients experienced grade 1 adverse reactions, specifically gastrointestinal irritation symptoms such as nausea and abdominal distension. These symptoms resolved upon discontinuation of ibuprofen.

## 4. Discussion

PDM, a widespread chronic pain disorder that affects many women of reproductive age and has a major negative effect on their quality of life. While Western medicine offers relief through NSAIDs and other medications, long-term use can lead to adverse effects like gastrointestinal symptoms and headaches. Therefore, there is a research focus on exploring safer, more effective, and side-effect-free treatment alternatives.

In this study, we chose acupuncture and moxibustion as a non-drug therapy for PDM. The choice of acupuncture and moxibustion in this study is rooted in their significant role within traditional Chinese medicine, supported by their efficacy in modern medical research. Specifically, the Huangdi internal needling method, derived from the Huangdi Neijing, holds a longstanding historical basis and widespread recognition for its therapeutic applications.^[18]^ After a 3-month treatment experiment, the therapeutic effect of the HIN group and the Ibuprofen group was significantly different, which suggested the advantages of acupuncture in treating PDM in many evaluation dimensions. The baseline characteristics of the 2 patient groups were comparable (no considerable differences), ensuring the objectivity and comparability of the experimental results. In terms of VAS scores, the pain intensity of dysmenorrhea in the HIN group progressively decreased from the first cycle to the third cycle, with sustained relief evident during the 6-month follow-up period posttreatment. The comparison was statistically significant involving the 2 groups, demonstrating that Huangdi’s internal acupuncture therapy not only alleviates symptoms but also provides enduring effects. Moreover, CMSS scores demonstrated notable improvement in menstrual-related symptoms within the HIN group. Through the safety evaluation of treatment, we observed that the Huangdi internal needle therapy did not cause any adverse events, which shows the safety of the therapy. This symptomatic relief is beneficial for enhancing patients’ quality of life. The ibuprofen regimen evaluated in this retrospective cohort reflected local clinical practice during the study period and may differ from standard guideline-recommended on-demand NSAID administration. Therefore, caution is required when generalizing the comparative findings to other treatment settings.

The long-term efficacy of treatment is a crucial finding in this study. Six months after treatment cessation, both the VAS and CMSS scores remained lower in the HIN group compared to the ibuprofen group, demonstrating the sustained therapeutic effect of Huangdi’s internal needling method. This is particularly significant for patients with PDM as it offers a potential long-term management strategy and reduces reliance on medications. Several studies suggest that Huangdi’s internal acupuncture therapy primarily treats PDM through several mechanisms: First, by regulating the endocrine system to reduce excessive prostaglandin secretion, thereby alleviating uterine contractions and pain.^[19]^ Second, it promotes pelvic blood circulation, enhances local blood supply, and relieves spasmodic pain. Additionally, acupuncture stimulates acupuncture points to trigger the release of natural pain-relieving substances such as endorphins, thereby increasing pain tolerance.^[20]^ Moreover, acupuncture regulates the autonomic nervous system and exhibits anti-inflammatory effects, effectively improving nervous dysfunction and inflammation associated with dysmenorrhea.^[21,22]^

However, this study also faces several limitations. First, this was a retrospective cohort study, and patients were grouped according to the actual treatment received rather than random allocation. Therefore, selection bias may have existed. For example, patients with more severe symptoms or a stronger preference for non-pharmacological therapy may have been more likely to choose acupuncture. Although baseline characteristics were comparable between groups, unmeasured confounders could still have influenced the results. Therefore, the present findings should be interpreted as associations rather than definitive causal evidence. Although the sample size was comparable to previous single-center observational studies, the relatively limited sample size may reduce statistical power and affect the generalizability of the findings.

In conclusion, this study provides evidence supporting the effectiveness and safety of Huangdi internal acupuncture for treating PDM. As a non-pharmacological intervention, acupuncture offers a viable and effective option for dysmenorrhea patients seeking alternative or adjunctive therapies. Future research can explore its potential applications in other chronic pain conditions.

## Author contributions

**Conceptualization:** Jing Liu, Wei Wang, Zun Liu.

**Data curation:** Jing Liu, Wei Wang, Zun Liu.

**Formal analysis:** Jing Liu, Wei Wang, Zun Liu.

**Funding acquisition:** Zun Liu.

**Writing – original draft:** Zun Liu.

**Writing – review & editing:** Zun Liu.
